# Deciphering the tumour microenvironment of clear cell renal cell carcinoma: Prognostic insights from programmed death genes using machine learning

**DOI:** 10.1111/jcmm.18524

**Published:** 2024-07-16

**Authors:** Hongtao Tu, Qingwen Hu, Yuying Ma, Jinbang Huang, Honghao Luo, Lai Jiang, Shengke Zhang, Chenglu Jiang, Haotian Lai, Jie Liu, Jianyou Chen, Liwei Guo, Guanhu Yang, Ke Xu, Hao Chi, Haiqing Chen

**Affiliations:** ^1^ Department of Urology Dazhou Central Hospital Dazhou Sichuan China; ^2^ School of Clinical Medicine The Affiliated Hospital, Southwest Medical University Luzhou China; ^3^ Three Gorges Hospital Chongqing University Chongqing China; ^4^ Department of Radiology Xichong People's Hospital Nanchong China; ^5^ Department of General Surgery Dazhou Central Hospital Dazhou China; ^6^ Department of Urology Dazhou Integrated Traditional Chinese Medicine and Western Medicine Hospital Dazhou Sichuan China; ^7^ Department of Urology The Dazhu County People's Hospital Dazhou China; ^8^ Department of Specialty Medicine Ohio University Athens Ohio USA; ^9^ Department of Oncology Chongqing General Hospital, Chongqing University Chongqing China

**Keywords:** cancer biomarkers, drug screen, machine learning, prognosis, programmed cell death, tumour microenvironment, urologic tumours

## Abstract

Clear cell renal cell carcinoma (ccRCC), a prevalent kidney cancer form characterised by its invasiveness and heterogeneity, presents challenges in late‐stage prognosis and treatment outcomes. Programmed cell death mechanisms, crucial in eliminating cancer cells, offer substantial insights into malignant tumour diagnosis, treatment and prognosis. This study aims to provide a model based on 15 types of Programmed Cell Death‐Related Genes (PCDRGs) for evaluating immune microenvironment and prognosis in ccRCC patients. ccRCC patients from the TCGA and arrayexpress cohorts were grouped based on PCDRGs. A combination model using Lasso and SuperPC was constructed to identify prognostic gene features. The arrayexpress cohort validated the model, confirming its robustness. Immune microenvironment analysis, facilitated by PCDRGs, employed various methods, including CIBERSORT. Drug sensitivity analysis guided clinical treatment decisions. Single‐cell data enabled Programmed Cell Death‐Related scoring, subsequent pseudo‐temporal and cell–cell communication analyses. A PCDRGs signature was established using TCGA‐KIRC data. External validation in the arrayexpress cohort underscored the model's superiority over traditional clinical features. Furthermore, our single‐cell analysis unveiled the roles of PCDRG‐based single‐cell subgroups in ccRCC, both in pseudo‐temporal progression and intercellular communication. Finally, we performed CCK‐8 assay and other experiments to investigate *csf2*. In conclusion, these findings reveal that *csf2* inhibit the growth, infiltration and movement of cells associated with renal clear cell carcinoma. This study introduces a PCDRGs prognostic model benefiting ccRCC patients while shedding light on the pivotal role of programmed cell death genes in shaping the immune microenvironment of ccRCC patients.

## 1 INTRODUCTION

Renal cell carcinoma (RCC) is a prevalent type of kidney cancer, originating in the renal epithelium and accounting for more than 90% of kidney cancer cases. This disease encompasses several histological and molecular subtypes, with clear cell renal cell carcinoma (ccRCC) being the most common and responsible for the majority of cancer‐related fatalities.^1^ The kidneys play a vital role in maintaining various physiological functions, including regulating electrolytes, water and acid–base balance, eliminating waste compounds and foreign substances, controlling blood pressure and secreting hormones.^2^

Treatment outcomes for patients with early‐stage localised ccRCC are favourable, with a 5‐year survival rate exceeding 90%. In contrast, patients with advanced metastatic disease face a significantly lower 5‐year survival rate of 12%.^3^ However, due to the aggressive and heterogeneous nature of ccRCC, patients still confront a relatively grim prognosis, with incidence and mortality rates steadily rising year by year.^4^ RCC is marked by numerous genetic abnormalities, and its clinical and biological diversity significantly influences drug resistance, signalling networks, distant metastasis and prognosis.^5^ Traditional clinical features like tumour grading and staging have proven insufficient in accurately predicting the progression and prognosis of ccRCC.^6^ Hence, there is an urgent demand for novel biomarkers to enhance the quality of life for ccRCC patients.

Programmed cell death is a highly regulated cellular process that typically occurs when cells sustain internal or external damage or when they need removal. The orchestrated clearance of unwanted, infected or potential tumour cells is governed by the programmed cell death pathway, underscoring its crucial role in maintaining homeostasis, defending the host against pathogens, cancer and various pathological conditions.^7^ Programmed cell death encompasses diverse forms, including apoptosis, pyroptosis, ferroptosis, autophagy, necroptosis, cuproptosis, parthanatos, entotic cell death, netotic cell death, lysosome‐dependent cell death, alkaliptosis, oxeiptosis, disulfidptosis, anoikis and efferocytosis, among others.

Numerous studies have already illustrated the close association of cell death‐related genes, such as copper death,^8^ iron death^9^ and proptosis,^10^ with the prognosis of ccRCC patients. Therefore, conducting a comprehensive investigation into apoptosis‐related genes and their connection with the prognosis and immune microenvironment of ccRCC patients holds significant clinical importance.

The evolving field of bioinformatics has underscored the growing significance of biomarkers in predicting cancer prognosis. Targeting programmed cell death for cancer therapy offers a promising avenue for improving patient outcomes. Programmed cell death mechanisms exert an influential role in shaping the response to immune checkpoint blockade and resistance in ccRCC by orchestrating the behaviour of mononuclear cells and modulating the levels of key mediators such as rTNF‐α or IL‐1β.^11^, ^12^ Nevertheless, the specific diagnostic and prognostic roles of Programmed Cell Death‐Related Genes (PCDRGs) in ccRCC remain insufficiently characterised. Thus, this investigation aims to comprehensively elucidate the intricate interplay between PCDRG expression patterns and the prognosis of ccRCC.

In the course of this study, we judiciously curated a panel of 17 dependable PCDRGs from the TCGA‐KIRC cohort. Subsequently, we formulated a robust prognostic model and devised a risk scoring system to scrutinise the intricate connections between PCDRGs, the immune microenvironment, immunotherapeutic responses and sensitivity to chemotherapy. Our principal objective is to underscore the invaluable role of PCDRGs in appraising the prognosis of ccRCC patients while simultaneously fostering the development of novel tools grounded in comprehensive genomic data analysis to refine and enhance therapeutic strategies.

Moreover, leveraging single‐cell sequencing data, our study conducted an in‐depth exploration of the complex intercellular communication networks and simulated gene expression patterns within the ccRCC microenvironment. This approach revealed previously undisclosed cellular‐level insights into the progression of the disease. The implications of our research are poised to furnish substantial academic underpinning for the advancement of tailored therapeutic and management approaches for ccRCC, with the ultimate goal of ameliorating patient prognosis and elevating the survival rates of individuals afflicted by ccRCC.

## 2 MATERIALS AND METHODS

### 2.1 Data collection and preprocessing

We acquired gene expression profiles and clinical records originating from 537 specimens of ccRCC and 60 specimens of normal tissue sourced from The Cancer Genome Atlas (TCGA) database (https://portal.gdc.cancer.gov/). Additionally, our dataset included 101 ccRCC samples downloaded from the arrayexpress (E‐MTAB‐1980) database (ebi.ac.uk/arrayexpress/). To ensure data consistency, we standardised each probe ID associated with genes into a gene symbol. In instances where a gene symbol corresponded to multiple probe IDs, we computed the gene's average expression value based on the mean expression values of the linked probe IDs. The set of 101 samples obtained from arrayexpress served as an independent validation dataset. As for single‐cell data, we accessed information from the Gene Expression Omnibus (GEO) database, particularly the GSE152938 dataset, encompassing GSM4630028 (PR1) and GSM4630031 (CT), and the GSE171306 dataset, comprising GSM5222644 (PR2). These datasets featured 10× single‐cell RNA sequencing (scRNA‐seq) data derived from two ccRCC patients and a single normal sample. Therefore, bulk and single‐cell data were obtained for subsequent analysis.

### 2.2 Marker genes were screened by single‐factor Cox regression and 10 kinds of machine learning

We sourced a comprehensive collection of 581 PCDRGs associated with apoptosis from the Import website (https://www.immport.org/resource) (Table S1). Subsequently, we leveraged R packages ‘limma’^13^ and ‘sva’ to introduce surrogate variables. These surrogate variables played a crucial role in estimating and mitigating batch effects in the gene expression data, as per the threshold criteria of logFC = 0.585 and adjusted *p* < 0.05, thereby enhancing the precision of our differential expression analysis. Further normalisation and scaling of the gene expression data were performed to account for technical variations among samples. This meticulous process culminated in the identification of 218 differential genes. After conducting the single‐factor Cox regression analysis, we identified the genes significantly associated with patient survival. Subsequently, we rigorously compared 101 distinct models generated by employing the 10 machine learning algorithms in both the training and validation sets. These machine learning algorithms encompassed the random survival forest,^14^ elastic network, Lasso, Ridge, stepwise Cox, CoxBoost, partial least squares regression for Cox, supervised principal components (SuperPC), generalised boosted regression modelling and survival support vector machine. Notably, it was revealed that a mere 17 genes were essential to achieve a satisfactory outcome within the Lasso+SuperPC model (Use Lasso for variable selection to reduce the number of predictors by selecting only the most relevant features. Apply SuperPC to the selected features from Lasso to capture the principal components (PCs) that are most predictive of the outcome). Consequently, we constructed a risk score based on these 17 carefully selected PCDRGs. The risk score for each patient (NRGs) was meticulously calculated using the following equation: Risk Score = ExpressionmRNA1 × CoefmRNA1 + ExpressionmRNA2 × CoefmRNA2 + … ExpressionmRNAn × CoefmRNAn.^15^ These risk scores were systematically computed for all ccRCC patients, we established the median for the training set. This enabled us to stratify ccRCC patients into low‐risk and high‐risk groups, for which survival curves were meticulously plotted. We also conducted Principal Component Analysis (PCA) employing the R package ‘limma’. Therefore, we obtained model genes through 10 machine learning.

### 2.3 Clinical feature validation of the model

In our comprehensive assessment, we meticulously conducted both single‐factor and multi‐factor Cox regression analyses to appraise the risk score as a truly independent prognostic factor. To gauge the model's predictive prowess, we harnessed sophisticated R packages, including ‘survival,’ ‘survminer’ and ‘timeROC’,^16^, ^17^ for the meticulous creation of Receiver Operating Characteristic (ROC) curves. These curves facilitated a detailed comparison of the model's performance in predicting the 1, 3 and 5‐year survival rates in conjunction with an array of clinical features. This robust validation protocol undeniably reaffirms the model's efficacy in harnessing clinical attributes to prognosticate patient outcomes. We validated the risk score as an independent prognostic factor using Cox regression analyses and ROC curves, confirming its efficacy in predicting survival rates.

### 2.4 Nomogram construction and independent prognostic analysis

In this rigorous analysis, we embarked upon the construction of a nomogram and, leveraging the sophisticated ‘rms’ R package, crafted an informative histogram that predominantly relied on the risk score in conjunction with an array of clinical‐pathological attributes. The overarching aim was to offer precise predictions for the 1, 3 and 5‐year survival rates of patients within the TCGA‐KIRC cohort.^17^ This nomogram stands as a powerful instrument, tailored for personalised prognosis analysis, amalgamating both clinical attributes and the risk score, thus enhancing the precision of survival forecasts. We developed a nomogram to integrate the risk score with clinical‐pathological attributes, enhancing the precision of 1, 3 and 5‐year survival predictions for TCGA‐KIRC patients.

### 2.5 Enrichment analysis

Our research endeavours encompassed a comprehensive enrichment analysis, facilitated by the robust ‘GSVA’ R package.^18^ Employing the ‘c2.cp.kegg. v7.4.symbols’ gene set from MSigDB, we meticulously conducted Gene Set Variation Analysis (GSVA). To pinpoint subgroup disparities of statistical significance, marked by an adjusted *p* < 0.05, we harnessed the analytical capabilities of the ‘limma’ R package. Our functional enrichment analysis ventured into an exploration of gene expression patterns pertaining to PCDRGs in the context of ccRCC. This extensive analysis encompassed the realms of functional annotations and enriched pathways. The investigation of Gene Ontology (GO) and Kyoto Encyclopedia of Genes and Genomes (KEGG) pathways (http://www.genome.jp/kegg/) was meticulously executed with the assistance of the ‘ClusterProfiler’ R package. Statistically significant distinctions were recognised when *p*‐values dipped below the threshold of 0.05. The fruits of this enrichment analysis have significantly deepened our understanding of the underlying biological processes (BP) and pathways intricately intertwined with PCDRGs in ccRCC.

### 2.6 Immune microenvironment analysis

In our quest to gauge the intricacies of immune infiltration, we harnessed the formidable CIBERSORT methodology, meticulously configuring the pFilter value to a precise 0.05. To ensure data consistency and uniformity, we deftly reshaped our dataset with the adept utilisation of the ‘limma’ and ‘reshape2’ R packages.^19^ Delineating the boundary between low‐risk and high‐risk patient groups was facilitated through the single‐sample Gene Set Enrichment Analysis technique. Our visualisation endeavours included the creation of informative boxplots that encapsulated vital insights into both immune cell populations and their functional manifestations, gracefully achieved with the assistance of the ‘ggpubr’ package. Furthermore, our quest for comprehensive insights extended to the acquisition of Tumour Immune Dysfunction and Exclusion (TIDE) data, meticulously sourced from http://tide.dfci.harvard.edu/ and tailored specifically to ccRCC. The profound statistical significance of our findings was underscored by a resounding *p*‐value of 9e‐13.

### 2.7 Drug sensitivity analysis

Our exploration into the realm of drug sensitivity embarked with a visit to the esteemed Cancer Genome of Drug Sensitivity (GDSC) database. This publicly accessible repository, as documented in the study by Yang et al.,^20^ provides valuable insights into molecular markers related to cancer cell drug sensitivity and therapeutic responses. Leveraging the extensive resources of the GDSC database, we embarked on an intricate inquiry into the intricate interplay between the expression profiles of the 17 carefully chosen PCDRGs and the critical parameter of half‐maximal inhibitory concentration (IC50) concerning a spectrum of anti‐tumour pharmaceuticals. Notably, our rigorous approach encompassed a stringent filtering criterion, firmly established at *p* < 0.001, ensuring the utmost precision and robustness of our findings. This in‐depth analysis serves as a valuable lens through which to fathom the profound implications of PCDRG expression on the sensitivity of cancer cells to an array of anti‐tumour medications.

### 2.8 Subsets processing of scRNA‐seq data

Our meticulous handling of the intricate scRNA‐seq data sets commenced with their transformation into Seurat objects, skillfully executed using the versatile R program ‘Seurat’ package.^21^ This initial step was closely followed by rigorous quality control (QC) procedures aimed at ensuring the data's integrity. The pivotal QC stage involved the meticulous examination of each cell's content, particularly focusing on the proportion of mitochondrial or ribosomal genes. Cells failing to meet the stringent quality criteria were systematically excluded from further analysis. Next, our dataset was subjected to a subsetting process that retained only cells with a gene count (referred to as nFeature_RNA) surpassing the threshold of 200 genes while ensuring they did not exceed 2500 genes. This selective approach allowed us to concentrate on a refined cell population, filtering out outliers and technical artefacts. Further refinement came in the form of identifying variable features, which represented genes with notable variability within the data set. The Variance Stabilizing Transformation method was judiciously applied to pinpoint the top 2000 variable features, emphasising those most pertinent to our analysis. In the pursuit of dimensionality reduction and the creation of a more manageable data representation, we harnessed the power of Uniform Manifold Approximation and Projection (UMAP) analysis. This technique effectively distilled the data into a lower‐dimensional space, rendering it amenable to intuitive visualisation and exploration. To gain insights into the diverse cellular landscape within the scRNA‐seq data, we judiciously employed the ‘SingleR’ package in R. This invaluable tool effectively identified various cell types, providing comprehensive clustering annotations as documented by Aran et al. in their study on lung single‐cell sequencing.^22^ This systematic annotation process not only elucidated the cellular diversity but also primed the data for further in‐depth analysis.

### 2.9 Temporal analysis and intercellular communication analysis

Our comprehensive exploration extended to temporal analysis and intercellular communication dynamics, harnessing advanced analytical tools to unveil intricate cellular pathways. For temporal analysis and cellular differentiation trajectory reconstruction, we harnessed the capabilities of the ‘Monocle’ R package. This sophisticated tool relies on gene counts and expressions to infer the trajectory of cellular differentiation. Employing the DDRTree method, we skillfully selected differentially expressed genes (DEGs)^23^ within the clustered results while simultaneously reducing the dimensionality of the data. To delve into intercellular communication networks, we availed ourselves of the rich resource known as the CellChatDB.human database. The process was further refined by the selective use of the ‘subsetDB’ function, which exclusively retained interaction data categorised as ‘Secreted Signalling’ from the extensive database. In our quest to unravel the complex web of cellular communication, ligands and receptors were strategically projected onto a protein–protein interaction network. Here, the ‘computeCommunProb’ function came into play, meticulously calculating communication probabilities. To visually encapsulate the intricate intercellular communication dynamics, we adroitly employed the ‘netVisual_circle’ function, creating a detailed network graph. This informative graph not only portrayed interaction counts but also conveyed communication weights and strengths. In pursuit of a more comprehensive overview, the ‘netVisual_aggregate’ function was judiciously employed, facilitating the generation of an aggregated network graph. Moreover, we harnessed the analytical power of the ‘netAnalysis_computeCentrality’ function to compute network centrality metrics, shedding light on the structural nuances and pivotal nodes within the intricate communication network.

### 2.10 Cell culture and transient transfection

The human ccRCC cell lines, namely 786‐O and Caki‐2, alongside the human renal proximal tubular epithelial cell line designated as HK‐2, were cultured in Dulbecco's modified Eagle's medium (DMEM, GIBCO). The culture medium was supplemented with 10% fetal bovine serum (FBS; Hyclone), 100 U/L penicillin and 100 mg/L streptomycin (Thermo Fisher). Standard incubation conditions were maintained at 37°C in an atmosphere enriched with 5% CO_2_. For transient transfection purposes, Lipofectamine 3000 (Invitrogen, Carlsbad, CA, USA) was employed following the manufacturer's recommended protocols. Specifically, this transfection method was utilised to introduce Negative Control (NC) and CDK6‐AS1 siRNA (RiboBio, Guangzhou, China) into colorectal carcinoma cells.

### 2.11 CCK‐8 assay

Cell viability was evaluated utilising the Cell Counting Kit‐8 (CCK‐8) assay. In a 96‐well plate, cells were initially seeded at a density of 1500 cells per well in 200 μL of complete medium, followed by an incubation period at 37°C. After each experimental manipulation, 20 μL of CCK‐8 reagent (Beyotime, Shanghai, China) was added to each well. Subsequently, the cells underwent an additional 2‐h incubation, and the optical density value (OD450 nm) was determined using a microplate reader.

### 2.12 Plate cloning experiment

In the context of a plate cloning experiment, the initial steps involve obtaining target cells from a culture vessel to assess their physiological robustness. Subsequently, these cells are dispersed into individual entities through a mild centrifugation process, ensuring that each clone originates from a single cell. The execution of the cloning process requires pre‐coating a Petri dish or culture plate with a growth medium containing the necessary constituents, establishing an optimal environment for cellular proliferation. Following the coating, dispersed cells are introduced onto the prepared medium at a density considered suitable for the intended purpose. The Petri dishes or plates are then placed within a cell culture incubator and cultivated under conditions maintaining an optimal temperature and controlled CO_2_ levels.

### 2.13 Wound healing experiment

To assess the migratory potential of renal clear cell carcinoma cells, a wound healing assay was utilised. Transfected cells, situated in six‐well plates, were subjected to incubation at 37°C until reaching approximately 80% confluence. Subsequently, precise wounds were created within the cell monolayer using a 200 μL sterile pipette tip. After the induction of wounds, cells underwent two rounds of rinsing with phosphate‐buffered saline to eliminate residual material. Following this, the culture medium was supplemented with serum‐free medium. The progression of cell migration into the wounded area was meticulously recorded using an Olympus inverted microscope at both the 0 and 24‐h time points.

### 2.14 Transwell assay

A quantity of 1 × 10^5^ cells was seeded into two distinct types of chambers, specifically Matrigel‐coated chambers (BD Biosciences, San Jose, CA) for the invasion assay and uncoated chambers for the migration assay. The upper chamber was filled with serum‐free medium, while the lower chamber was supplied with complete DMEM medium. After a 24‐h incubation period, cells that effectively migrated or invaded through the membrane were meticulously fixed using a 4% paraformaldehyde solution and subsequently stained with 0.1% crystal violet. Quantification of these cellular events was executed using an optical microscope (Thermo Fisher, Waltham, MA, USA).

### 2.15 Statistical analysis

Statistical analyses were conducted using R software version 4.3.1 and Strawberry Perl version 5.32.1. The comparative assessment of overall survival (OS) between high‐risk and low‐risk cohorts was accomplished through Kaplan–Meier (KM) survival plots, accompanied by the utilisation of the log‐rank test. Predictive capabilities of correlation features, derived from the LASSO Cox regression model, were assessed via ROC curves. The distinction in the representation of tumour‐infiltrating immune cells, immune checkpoints and immune functionality between the two cohorts was explored employing the Wilcoxon test. Statistical significance was acknowledged when *p*‐values were <0.05 and the False Discovery Rate (FDR) remained below 0.05. Analysis of the results from the CCK‐8 assay was performed using GraphPad Prism Software (version 8.3.0). The data are presented as means with corresponding standard deviations and were derived from three independent experiments. The statistical evaluation was carried out using analysis of variance. Significance was declared when the *p*‐value was <0.05.

## 3 RESULTS

### 3.1 Identification of differentially expressed PCDRGs

**FIGURE 1:**
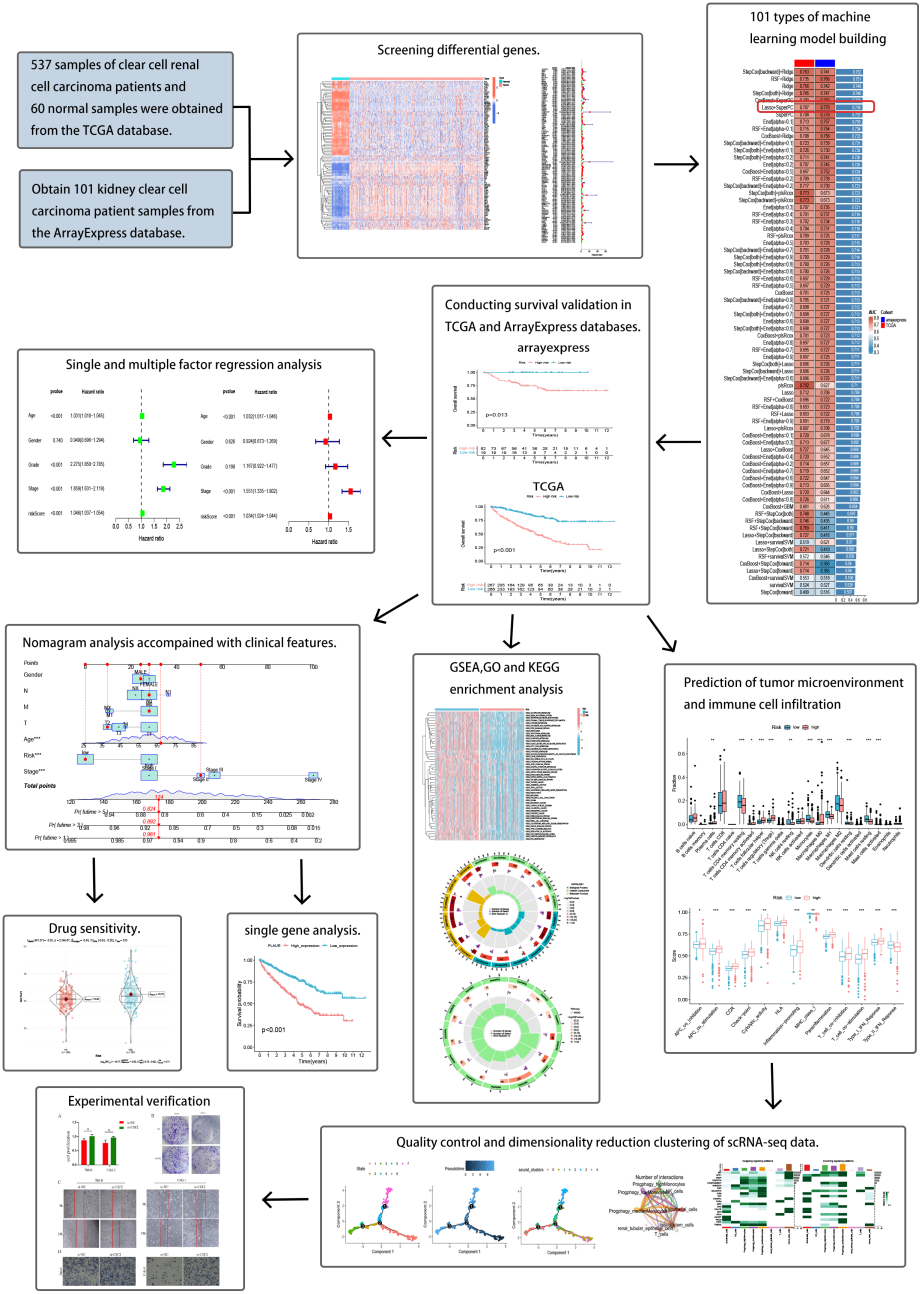
The main ideas and methods of this study.

**FIGURE 2:**
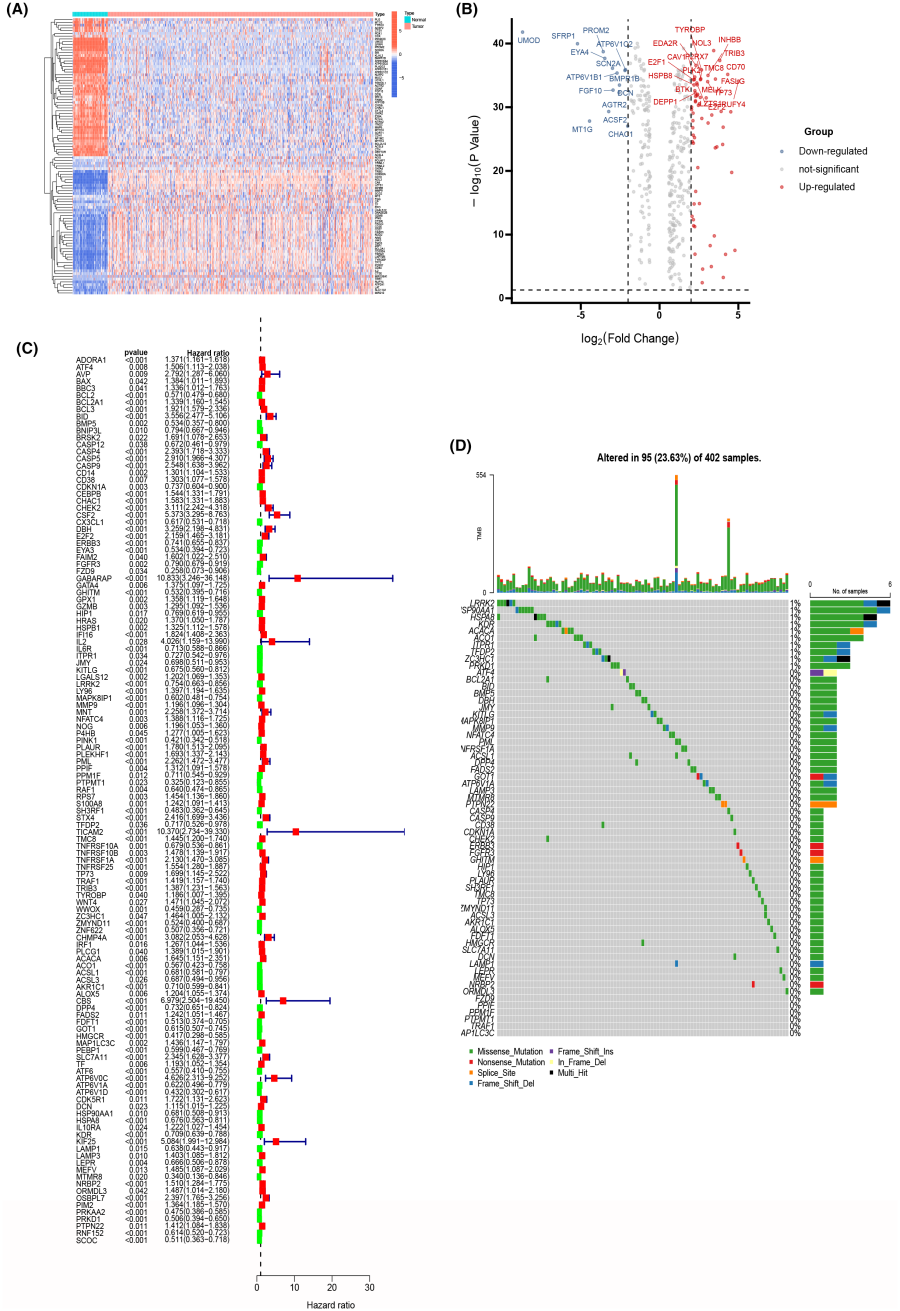
Differential analysis was performed to select alternative programmed cell death‐related genes (PCDRGs). (A, B) Heatmaps and volcano plots of differential PCDRGs. (C)Single‐factor Cox regression analysis found that PCDRGs was correlated with OS (*p* < 0.05). (D)The waterfall map was obtained by mutation analysis of 127 genes.

To elucidate our research methodology, we offer a schematic representation of the primary investigative approach and design steps (Figure 1). Our initial step involved acquiring gene expression profiles, encompassing 537 ccRCC samples, and 60 normal samples from TCGA. Employing the ‘limma’ package in R, we embarked on a differential gene expression analysis within the ccRCC cohort, culminating in the identification of 387 DEGs that exhibited a minimum fold change of 1.5. This distinctive set comprised 133 upregulated PCDRGs and 255 downregulated PCDRGs, effectively portraying the regulatory landscape of PCDRGs in ccRCC. Intriguingly, we harnessed the potential of the ‘pheatmap’ package to meticulously visualize our results, generating compelling heatmaps and illuminating volcano plots (Figure 2B) that eloquently portrayed the distinctive expression patterns of PCDRGs. Subsequently, we undertook a single‐factor Cox regression analysis, leveraging the ‘survival’ and ‘survminer’ packages, and scrutinized the influence of 218 differentially expressed PCDRGs on OS. The analysis underscored the statistical significance of PCDRGs in the context of patient prognosis (*p* < 0.05) (Figure 2C). Mutations of 127 genes were analysed and the results were visualized using the ‘maftools’ package to generate a waterfall map (Figure 2D), elucidating the intricate mutational landscape, thereby deepening our understanding of the differential expression of PCDRGs and their profound connection with OS in ccRCC patients.

### 3.2 Ten machine learning algorithms for model selection and survival validation

**FIGURE 3:**
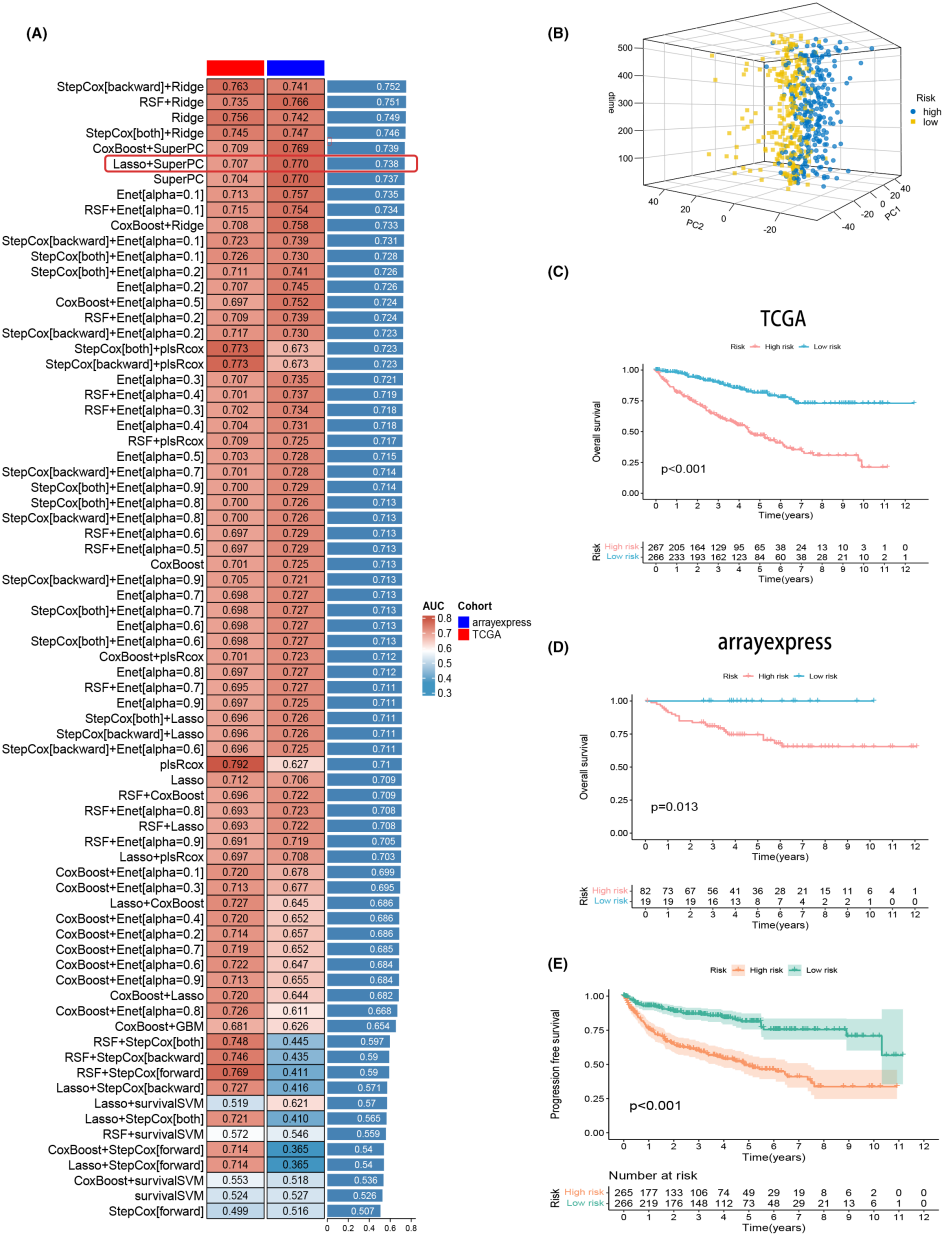
We applied 10 different machine learning algorithms to construct 101 models in total and selected the Lasso+SuperPC combination model, Risk models for 17 PCDRGs were obtained (A). PCA plots showed significant differences between groups (B). Survival analysis verified that the prognostic differences between the training group (C) and the test group (D) were statistically significant (*p* < 0.05). Survival validation from progression‐free survival showed significant prognostic differences (*p* < 0.05) (E).

We obtained 101 samples from the ArrayExpress database and combined them with the training set. We applied 10 different machine learning algorithms to construct 101 models in total. Model performance was assessed using the C‐index, and the Lasso+SuperPC combination model was selected. This model consisted of 17 PCDRGs, namely *bcl2*, *bcl3*, *bid*, *cdkn1a*, *chac1*, *csf2*, *cx3cl1*, *bdh*, *ddias*, *erbb3*, *il4*, *ltbr*, *pdcd5*, *pink1*, *plaur*, *ybx3* and *prkag3*. Although the AUC values of the model combinations ranked earlier are relatively high, they require tens or even hundreds of genes for fitting. In contrast, this model combination achieves satisfactory performance with only 17 genes. The prognostic indices for these genes were determined and shown as follows: −3.555906031, 3.613612742, 2.701442475, −1.72130997, 2.687054603, 1.333552089, −3.69047182, 1.88604489, 1.469790779, −3.014101244, 1.233643483, 1.813817952, 3.27786348, −4.146124763, 4.005120164, 2.348694954 and 0.014816641 (Figure 3A). Based on the prognostic indices, we divided both the training and validation sets into high‐risk and low‐risk groups using the median value from the training set. Analysis was performed using the ‘limma’ and ‘ggplot2’ packages, and PCA plots showed significant differences between the groups (Figure 3B). Survival analysis confirmed statistically significant prognostic differences in the training set (Figure 3C) and validation set (Figure 3D) (*p* < 0.05). Additionally, survival validation based on progression‐free survival also demonstrated statistically significant prognostic differences (*p* < 0.05) (Figure 3E).

### 3.3 Validation of the risk model across diverse clinical parameters

**FIGURE 4:**
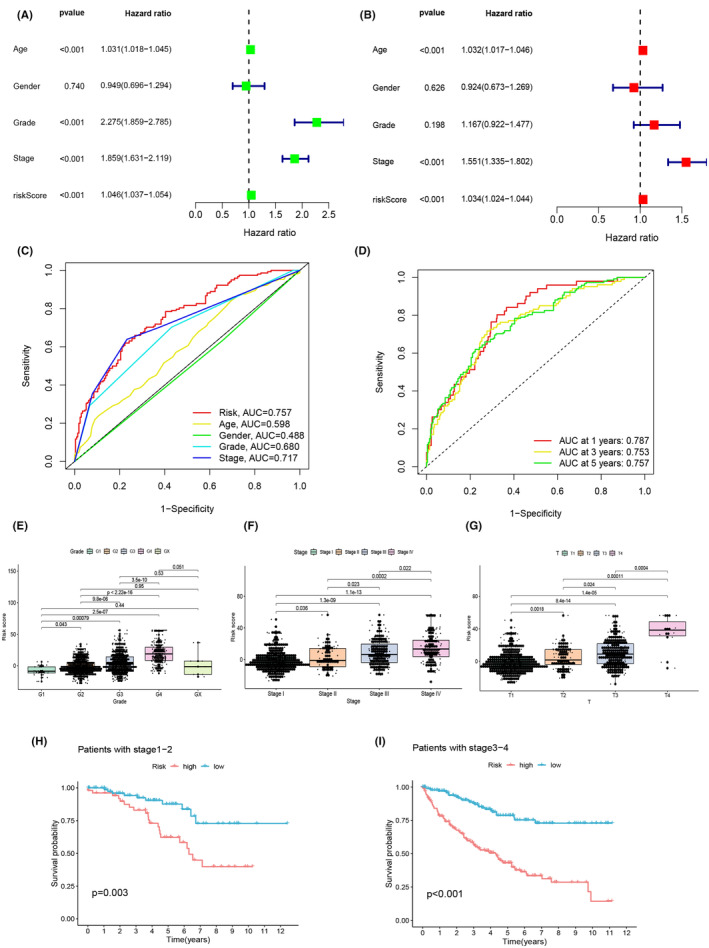
(A) Univariate cox analysis showed that ccRCC risk score was significantly associated with prognosis (*p* < 0.001). (B) Multivariate cox analysis showed that risk score (*p* < 0.001) were independent and reliable predictors of outcome risk. (C) ROC curves for different clinical characteristics. (D) We plotted ROC curves at 1, 3 and 5 years (AUC = 0.787, 0.753, 0.757). (E–G) Boxplots for grade, stage, and T stage were plotted. (H, I) We plotted survival curves for T stage (T1‐2 and T3‐4) analysis of the entire sample subgroup.

In the pursuit of establishing the risk model's resilience concerning its associations with key prognostic determinants, we conducted an extensive battery of statistical tests. We integrated the OS data of patients afflicted by ccRCC with their clinical attributes, embarking on a comprehensive exploration into the prognostic potential of the 17 PCDRG‐derived risk model. The findings were notably robust. Single‐factor Cox analysis vividly illuminated a strong and statistically significant correlation between ccRCC risk scores and patient prognosis (*p* < 0.001), as depicted in Figure 4A. Subsequently, our meticulous multi‐factor Cox analysis reaffirmed the risk score's status as a self‐sufficient and reliable prognostic indicator, thus substantiating its independence (*p* < 0.001), as visualized in Figure 4B. In tandem with these discoveries, single‐factor Cox analysis pinpointed age, grade, and stage as prominent predictors significantly associated with prognosis (*p* < 0.001). Correspondingly, multi‐factor Cox analysis reiterated the importance of age and stage in shaping prognostic landscapes (*p* < 0.001). In the domain of ROC curve analysis, the risk score unequivocally emerged as the most potent predictive element amidst the diverse clinical parameters (Figure 4C). To further enhance predictive capabilities, ROC curves were charted for 1‐, 3‐ and 5‐year survival horizons. The outcomes underscored exceptional sensitivity and specificity, encapsulated by impressive AUC values of 0.787, 0.753 and 0.757, respectively (Figure 4D), validating the risk score's prowess as a discerning tool for assessing survival prospects. A subsequent phase of our analysis ventured into clinical correlation evaluations, effectively executed through the ‘limma’ and ‘ggpubr’ packages. Exploring the parameters of grade, stage, and T stage, these assessments took the form of expressive box plots (Figure 4E–G). The dynamic patterns revealed a proportional elevation in risk as grade, stage, and T stage advanced. Strikingly, this escalation yielded significant differences across each subgroup, reinforcing the statistical significance (*p* < 0.05). Refining our scrutiny through subgroup analysis, we focused on the T stage subcategories (T1‐2 and T3‐4). The results poignantly demonstrated the notably shorter survival durations experienced by high‐risk patients compared to their low‐risk counterparts (Figure 4H, I). Collectively, these results are emblematic of the risk model's capacity to effectively prognosticate ccRCC within specific clinical contexts and their respective subgroups. The evident superiority in clinical decision‐making, as evidenced by the highest net benefit, reinforces the influence of the PCDRG‐based risk model over traditional counterparts.

### 3.4 Nomogram development for enhanced clinical utility of the risk model

**FIGURE 5:**
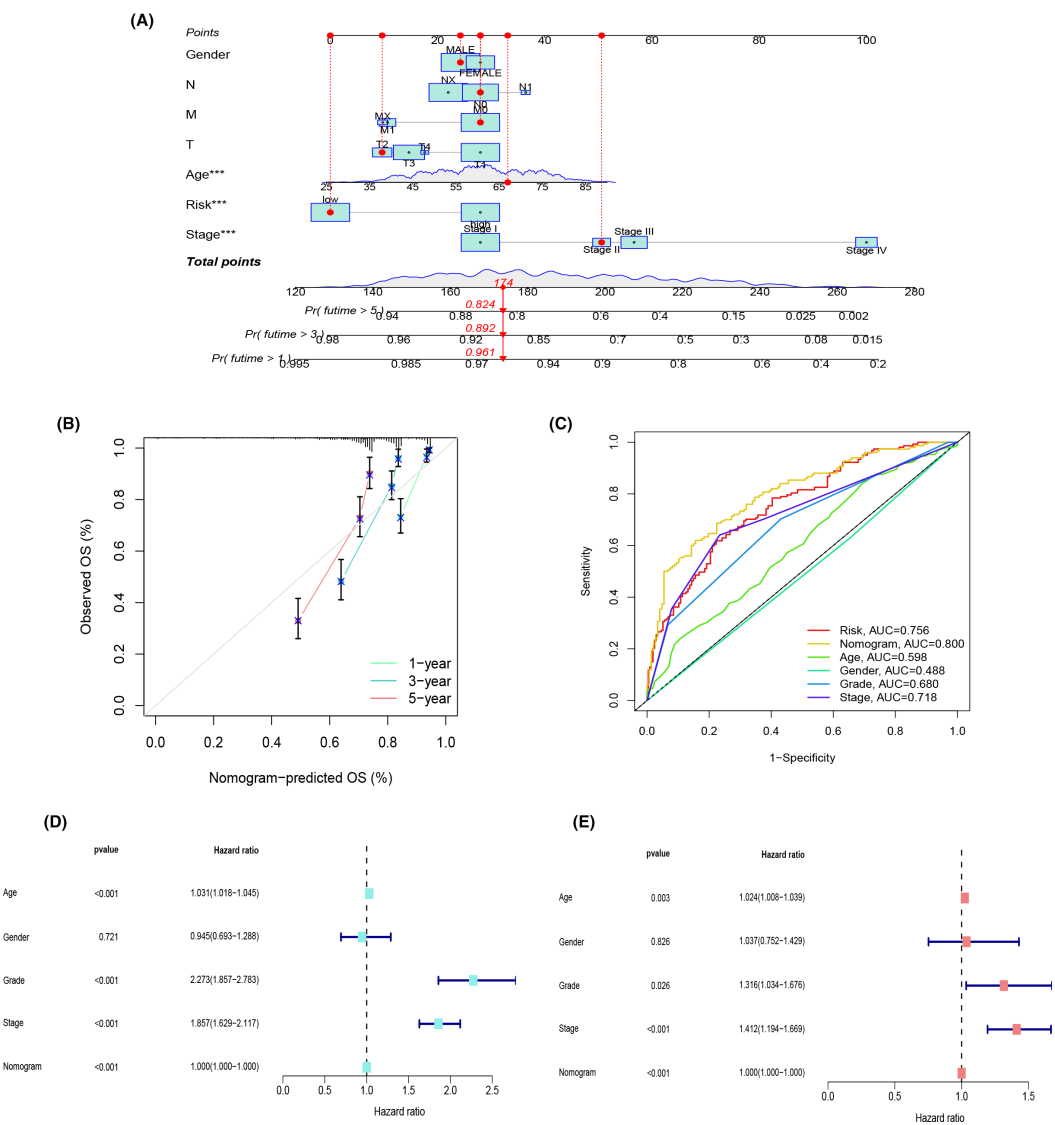
We constructed a Nomogram to expand the clinical application and usability of the constructed risk model. (A) We constructed a nomogram based on gender, N stage, M stage, age, T stage, risk score, and stage to predict the prognostic survival probability at 1, 3 and 5 years for ccRCC patients. (B) Calibration curves at 1, 3 and 5 years. (C) ROC curves for risk score, nomogram, age, gender, grade, and stage. (D, E) Univariate and multivariate Cox analyses were performed to compare the nomogram model with other clinical factors.

In the pursuit of providing a comprehensive prognostic support system for clinical decision‐making, we have designed a nomogram that integrates several key clinical parameters. This nomogram is rooted in gender, N stage, M stage, age, T stage, risk score, stage and grade, offering a visual representation to predict the 1‐, 3‐ and 5‐year survival probabilities of ccRCC patients (Figure 5A).To ensure the reliability of this predictive tool, we conducted calibration assessments for 1, 3 and 5‐year OS. These evaluations yielded calibration curves that distinctly illustrated the alignment between projected and observed outcomes, signifying a high level of consistency (Figure 5B). Of notable significance were the factors of age, risk score, and stage, each demonstrating considerable statistical relevance (*p* < 0.05). Consequently, we executed receiver operating characteristic (ROC) curve analyses, comparing the predictive potential of the nomogram alongside clinical attributes such as risk score, age, gender, grade, and stage. This comprehensive evaluation revealed the nomogram as the most powerful predictor (AUC = 0.800) when contrasted with other clinical characteristics, outperforming risk score (AUC = 0.756) and the remaining variables (Figure 5C). Further validation via single‐factor (Figure 5D) and multi‐factor Cox analyses (Figure 5E) reinforced the significance of the nomogram model in relation to OS among ccRCC patients. These findings underscore the nomogram's value as an indispensable tool for enhancing the accuracy of survival predictions and bolstering clinical decision‐making for individuals affected by ccRCC.

### 3.5 Enrichment analysis of model‐associated genes

**FIGURE 6:**
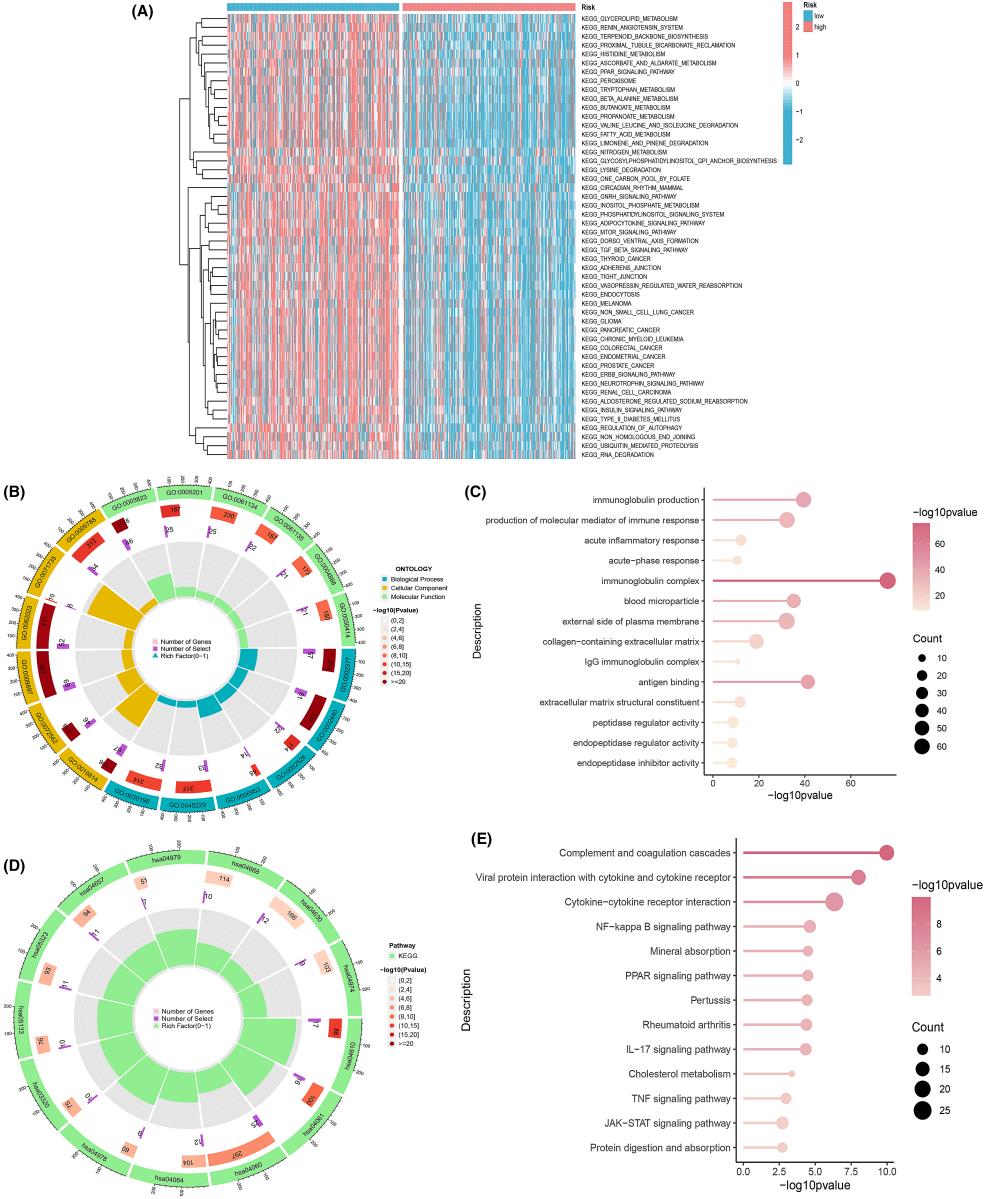
We performed enrichment analysis on the model 17‐ PCDRGs. (A) We performed an enrichment analysis for the model genes. Heatmap of GSEA enrichment analysis. (B, C) The GO enrichment and (D, E) the KEGG enrichment.

To delve deeper into the genomic distinctions between high‐risk and low‐risk subgroups, we harnessed the power of GSVA to scrutinize alterations in the activity of gene sets within diverse pathways. This investigation uncovered 122 notably enriched pathways, encompassing prominent entries like ‘KEGG_LIMONENE_AND_PINENE_DEGRADATION’, ‘KEGG_VALINE_LEUCINE_AND_ISOLEUCINE_DEGRADATION’ and ‘KEGG_RIBOSOME’, to name a few. These insights were presented visually through a heatmap, elucidating the disparities (Figure 6A). In parallel, we embarked on an exploration of KEGG and GO enrichment analyses to appraise the disparities within gene sets between the high‐risk and low‐risk subgroups. Our stringent criteria for significance were defined as FDR < 0.05 and *p* < 0.05, a selective approach that brought forth significant findings. This multifaceted endeavour shed light on the enrichment of BP, cellular components (CC), molecular functions (MF), and the pertinence of signalling pathways to the risk score. The GO enrichment segment uncovered a myriad of BP that stood out, including but not limited to immunoglobulin production, production of molecular mediators involved in immune response, acute inflammatory response and related facets (Figure 6B,C). The CC portion was marked by enrichments in categories like immunoglobulin complexes, blood microparticles, the extracellular side of the plasma membrane, collagen‐containing extracellular matrix and IgG immunoglobulin complexes. Meanwhile, the MF module highlighted significant roles in antigen binding, extracellular matrix structural constituents, peptidase regulator activity, endopeptidase regulator activity and endopeptidase inhibitor activity. The KEGG enrichment analysis expanded our horizons into pathways encompassing complement and coagulation cascades, viral protein interaction with cytokine and cytokine receptor, cytokine‐cytokine receptor interaction, NF‐kappa B signalling pathway, mineral absorption, PPAR signalling pathway, pertussis, rheumatoid arthritis, IL‐17 signalling pathway and others (Figure 6D,E).These comprehensive findings underscore a profound link between the enrichment analysis and immune responses. Subsequently, we embarked on an exhaustive examination of the immune landscape within the two distinct subgroups of ccRCC patients.

### 3.6 Tumour microenvironment and immune cell infiltration prediction

**FIGURE 7:**
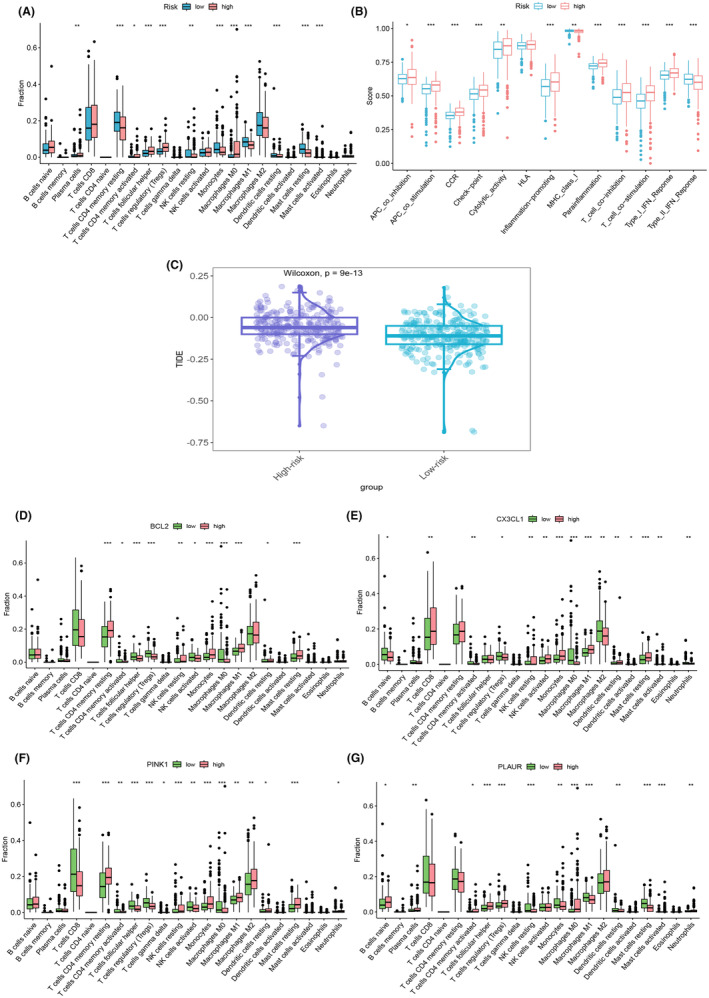
Prediction of tumour microenvironment and immune cell infiltration using model 17‐ PCDRGs. (A) Box plot of immune cells. (B) Box plot of immune function. (C)The TIDE of risk models for 17‐PCDRGs (*p* = 9e‐13). (D–G) The immune cell infiltration analysis of single genes, such as *bcl2*, *bcl3*, *pink1* and *plaur*, showed significant differences between the high and low risk groups in monocytes *p < 0.05; **p < 0.01; ***p < 0.001.

Utilizing the advanced CIBERSORT algorithm, we embarked on an intricate exploration of immune cell infiltration levels within each sample. Our meticulous examination unveiled compelling disparities in immune cell infiltration between the high‐risk and low‐risk groups. The comprehensive analysis extended to various subtypes of T cells, monocytes, and macrophages, revealing discernible distinctions. Notably, the low‐risk group exhibited heightened infiltration levels in T cells CD4 memory resting, macrophages M1, and monocytes. In contrast, the high‐risk group demonstrated elevated infiltration levels in T cells follicular helper, regulatory T cells (Tregs), and macrophages M0 (Figure 7A). The depth of our analysis did not stop at cellular infiltration alone; we ventured into an exploration of immune function disparities between the high‐risk and low‐risk groups. Apart from shedding light on MHC class I and Type II IFN Response, our findings underscored that the high‐risk group boasted superior immune infiltration levels in comparison to the low‐risk group (Figure 7B).Moreover, we harnessed the power of TIDE scores, gleaned from the TIDE website (http://tide.dfci.harvard.edu/), and combined these scores with the PCDRGs risk model. Our data unveiled significant disparities between the high‐risk and low‐risk groups (*p* = 9e‐13). Notably, the high‐risk group was associated with a heightened risk of immune escape relative to their low‐risk counterparts (Figure 7C).In a bid to further validate the model genes' potential in predicting immune cell infiltration, we conducted a granular analysis of single‐gene immune cell infiltration. This deep dive highlighted significant disparities in the infiltration levels of monocytes between the high‐risk and low‐risk groups for the model genes *bcl2* (Figure 7D), *bcl3* (Figure 7E), *pink1* (Figure 7F) and *plaur* (Figure 7G). These findings accentuate the crucial role of the model genes in dictating immune cell infiltration dynamics.

### 3.7 Drug sensitivity assessment

**FIGURE 8:**
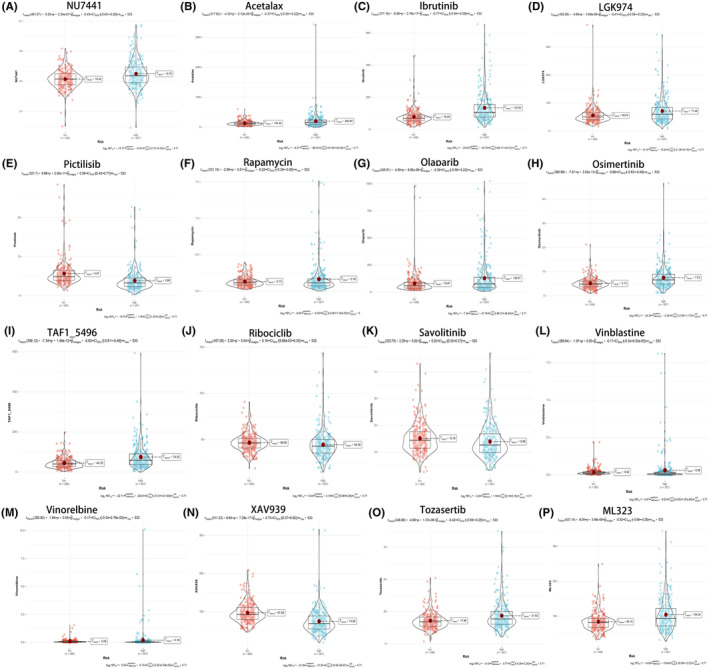
We performed susceptibility analyses for individual PCDRGs and plotted boxplots of their results. In (A) nu7441, (B) acetalax, (C) ibrutinib, (D) lgk974, (E) pictilisib, (F) rapamycin, (G) olaparib, (H) osimertinib, (I) taf1_5496, (J) ribociclib, (K) savolitinib, (L) vinblastine, (M) vinorelbine, (N) xav939, Significant differences between the high‐risk and low‐risk groups were observed in susceptibility analysis for both (O) tozasertib and (P) ml323 (*p* < 0.05).

To delve into the intricate interplay between high‐risk and low‐risk cohorts and their respective responsiveness to therapeutic agents, we conducted a thorough evaluation of 16 drugs pertinent to ccRCC. Harnessing the comprehensive insights derived from our risk scores and the expression patterns of the 17 PCDRGs, we meticulously constructed a series of enlightening violin plots, each offering a detailed view of the drug‐specific profiles. These encompassed nu7441 (Figure 8A), acetalax (Figure 8B), ibrutinib (Figure 8C), lgk974 (Figure 8D), pictilisib (Figure 8E), rapamycin (Figure 8F), olaparib (Figure 8G), osimertinib (Figure 8H), taf1_5496 (Figure 8I), ribociclib (Figure 8J), savolitinib (Figure 8K), vinblastine (Figure 8L), vinorelbine (Figure 8M), xav939 (Figure 8N), tozasertib (Figure 8O) and ml323 (Figure 8P).Our meticulous analysis brought to the forefront significant disparities in the responsiveness to several of these drugs between the high‐risk and low‐risk groups (*p* < 0.05). This compelling revelation underscores the pivotal clinical implications of our 17 PCDRGs risk model, not only as a prognostic tool but also as a guiding light for the evaluation of drug sensitivity, thereby aiding in the formulation of tailored treatment strategies for patients grappling with ccRCC.

### 3.8 Kaplan–Meier (KM) survival curves for individual PCDRGs

**FIGURE 9:**
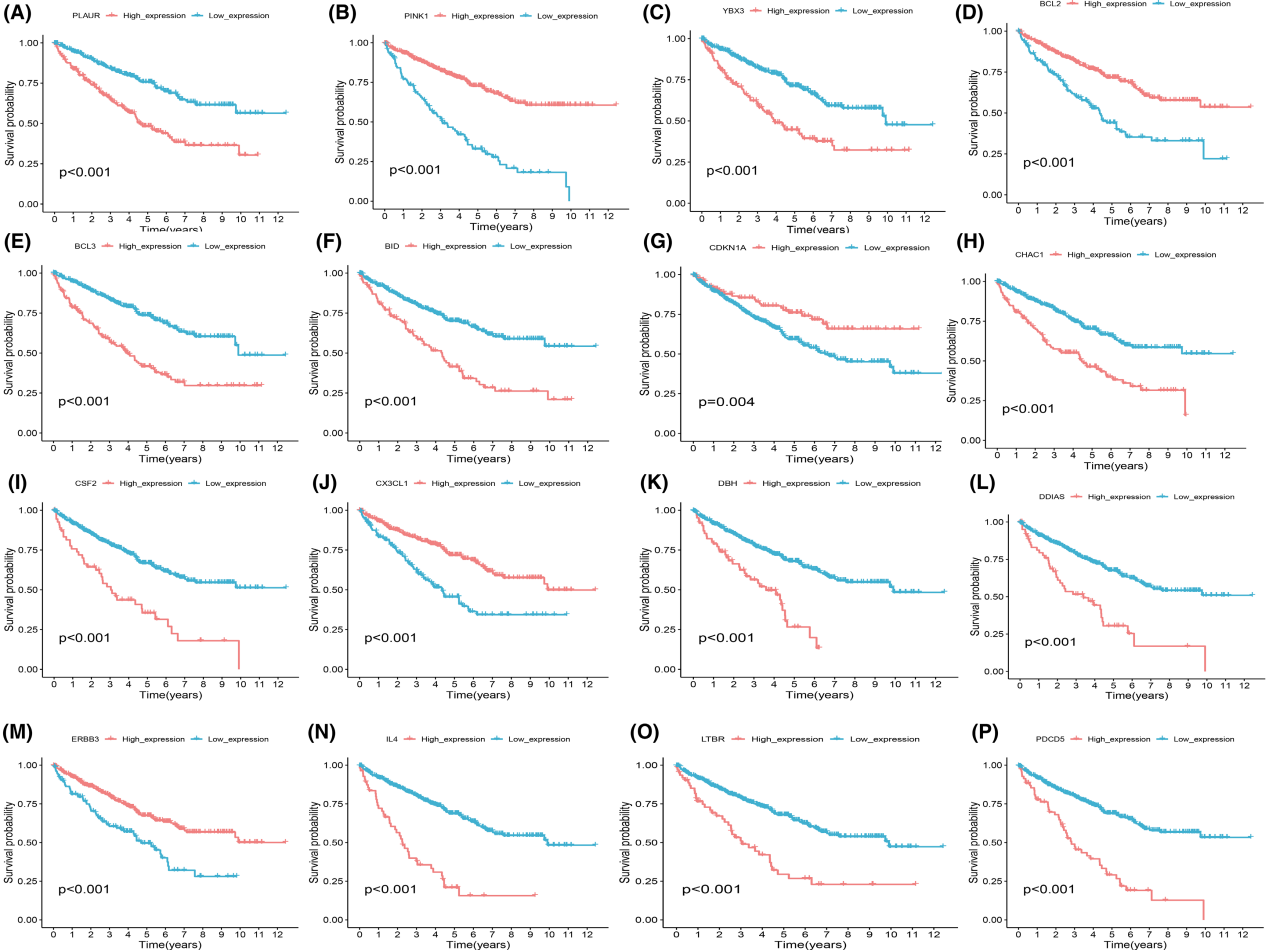
We plotted KM risk curves for individual PCDRGs. We plotted KM survival curves for individual PCDRGs, including (A) *plaur* (*p* < 0.001), (B) *pink1* (*p* < 0.001), (C) *ybx3* (*p* < 0.001), (D) *bcl2* (*p* < 0.001), (E) *bcl3* (*p* < 0.001), (F) *bid* (*p* < 0.001), (G) *cdkn1a* (*p* = 0.004), (H) *chac1* (*p* < 0.001), (I) *csf2* (*p* < 0.001), (J) *cx3cl1* (*p* < 0.001), (K) *bdh* (*p* < 0.001), (L) *ddias* (*p* < 0.001), (M) *erbb3* (*p* < 0.001), (N) *il4* (*p* < 0.001), (O) *ltbr* (*p* < 0.001) and (P) *pdcd5* (*p* < 0.001).

In order to ascertain the independent prognostic value of each of the PCDRGs with respect to OS, a comprehensive survival analysis was meticulously carried out. These analyses were predicated on the distinctive risk scores associated with each PCDRG. The results were elegantly depicted through the construction of KM survival curves. Impressively, all 17 of the PCDRGs under scrutiny unequivocally demonstrated a statistically significant predictive accuracy in terms of OS (*p* < 0.05).This select group of individual PCDRGs encompasses *plaur* (*p* < 0.001) (Figure 9A), *pink1* (*p* < 0.001) (Figure 9B), *ybx3* (*p* < 0.001) (Figure 9C), *bcl2* (*p* < 0.001) (Figure 9D), *bcl3* (*p* < 0.001) (Figure 9E), *bid* (*p* < 0.001) (Figure 9F), *cdkn1a* (*p* = 0.004) (Figure 9G), *chac1* (*p* < 0.001) (Figure 9H), *csf2* (*p* < 0.001) (Figure 9I), *cx3cl1* (*p* < 0.001) (Figure 9J), *bdh* (*p* < 0.001) (Figure 9K), *ddias* (*p* < 0.001) (Figure 9L), *erbb3* (*p* < 0.001) (Figure 9M), *il4* (*p* < 0.001) (Figure 9N), *ltbr* (*p* < 0.001) (Figure 9O), *pdcd5* (*p* < 0.001) (Figure 9P) and *prkag3* (*p* = 0.007). These findings attest to the robust individual prognostic potential of these PCDRGs in the context of ccRCC.

### 3.9 Quality control and dimension reduction clustering of scRNA‐seq data

**FIGURE 10:**
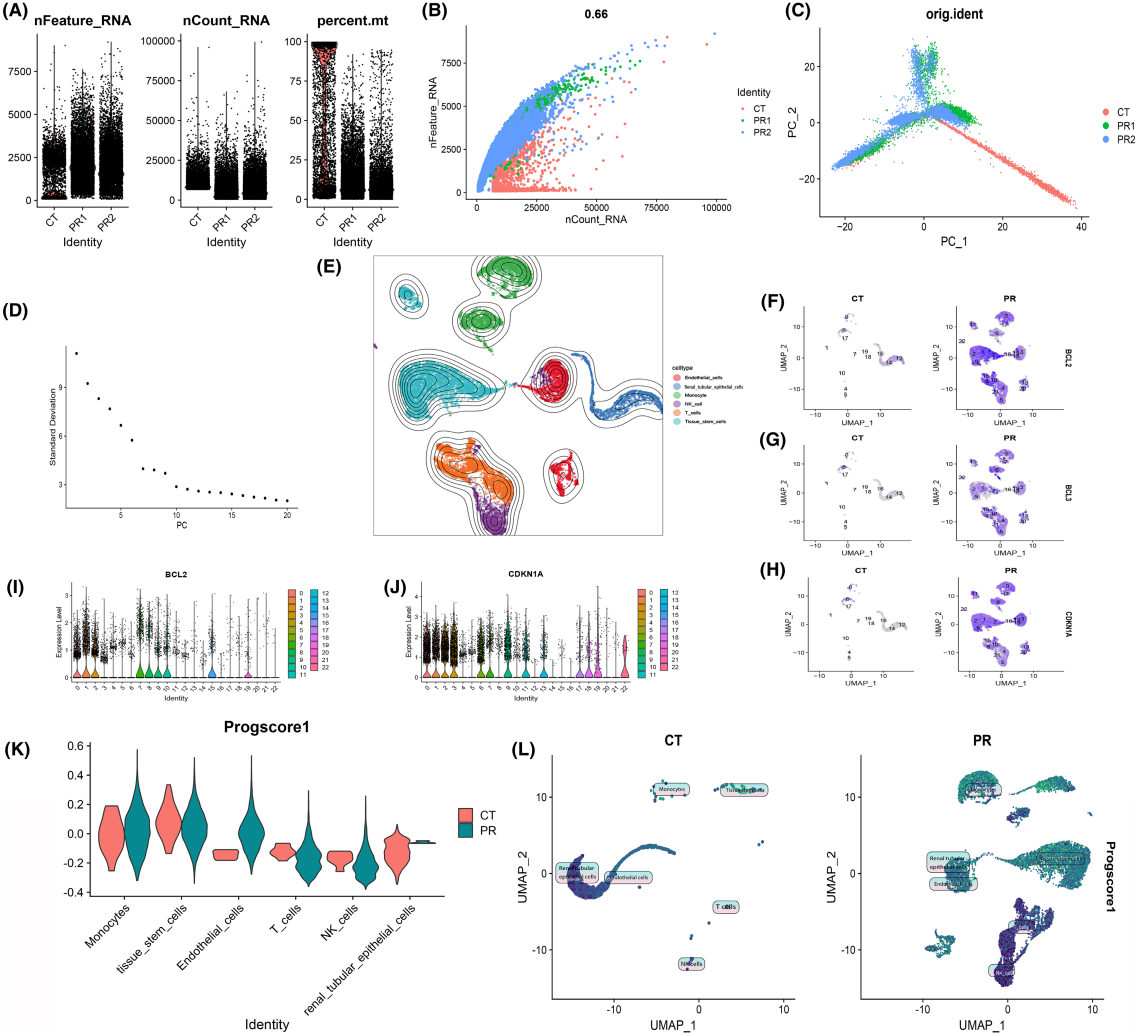
We performed quality control on scRNA‐seq data with dimensionality reduction clustering. (A) We preprocessed and normalized the single‐cell data. (B) Scatter plot of the correlation between nCount and nFeature. (C, D) We selected the data from the top 10 PCS for downsizing. (E) We grouped by ‘seurat_clusters’ and plotted the UMAP. (F–H) We plotted the expression of ‘*bcl2*’, ‘*bcl3*’ and ‘*cdkn1a*’ in cell clusters using umap (CT refers to normal samples, while PR refers to samples of clear cell renal cell carcinoma.). (I, J) We plotted the violin plot of ‘*bcl2*’ and ‘*cdkn1a*’ in each cell cluster. (K) Violin plot of the expression of various cells. (L) Comparison of umap between disease group and control group.

Our exploration of gene expression profiles at the single‐cell level necessitated meticulous preprocessing and data normalization (Figure 10A). A crucial aspect of this procedure involved assessing cell quality through rigorous scrutiny of unique molecular identifier (UMI) and gene‐related metrics. A robust and high‐quality dataset was ascertained based on the compelling correlation observed between the number of UMI and the number of unique genes (nCount and nFeature) (*r* = 0.66) (Figure 10B).To unveil the intrinsic structure of the single‐cell data, PCA was meticulously executed, employing the RunPCA function encapsulated within the Seurat package. The data were processed to include only the most highly variable genes, with subsequent scaling through the ‘scaledata’ function. The determination of anchor points was a pivotal step in reducing dimensions via PCA, with data from the top 10 PCs being judiciously retained for downstream analyses (Figure 10C, D).The cells were thoughtfully stratified based on the ‘seurat_clusters’ and artfully visualized using UMAP (Figure 10E). For a more granular understanding of the gene expression landscapes, UMAP plots were ingeniously harnessed to illustrate the expression patterns of ‘*bcl2*’ (Figure 10F), ‘*bcl3*’ (Figure 10G) and ‘*cdkn1a*’ (Figure 10H) within distinct cellular clusters. Further elucidating the intricacies of gene expression, violin plots were generated to delineate the expression profiles of PCDRGs across various cell clusters. Notably, ‘*bcl2*’ (Figure 10I) and ‘*cdkn1a*’ (Figure 10J) exhibited elevated expression levels in clusters 0, 6, 11, 17 and 12. To impart functional relevance to these findings, the 17 PCDRGs were systematically scored across cell types, and their OS scores were diligently computed. The analysis underscored the heightened expression of the 17 PCDRGs within immune cell subtypes localized at sites of infection in comparison to the control group. Specifically, Renal tubular epithelial cells, NK cells, monocytes and T cells prominently featured heightened expression of these PCDRGs (Figure 10K). Subsequently, we engaged the Seurat package's findclusters function to meticulously categorize cells, with the singleR package enabling robust cell type annotation. This effort culminated in the recognition of five discernible cell types: Monocyte, Renal tubular epithelial cells, Tissue stem cells, NK cells and Endothelial cells (Figure 10L). A compelling discovery emerged from the pronounced variations in PCDRG expression within monocytes when juxtaposing the high‐risk and low‐risk groups. This observation strongly resonated with our prior findings from Figure 7D (*bcl2*), Figure 7E (*bcl3*), Figure 7F (*pink1*) and Figure 7G (*plaur*). In summation, the spotlight of our ongoing investigations converged on monocytes for further in‐depth exploration.

### 3.10 Single monocyte cell pseudo‐time analysis and cell communication

**FIGURE 11:**
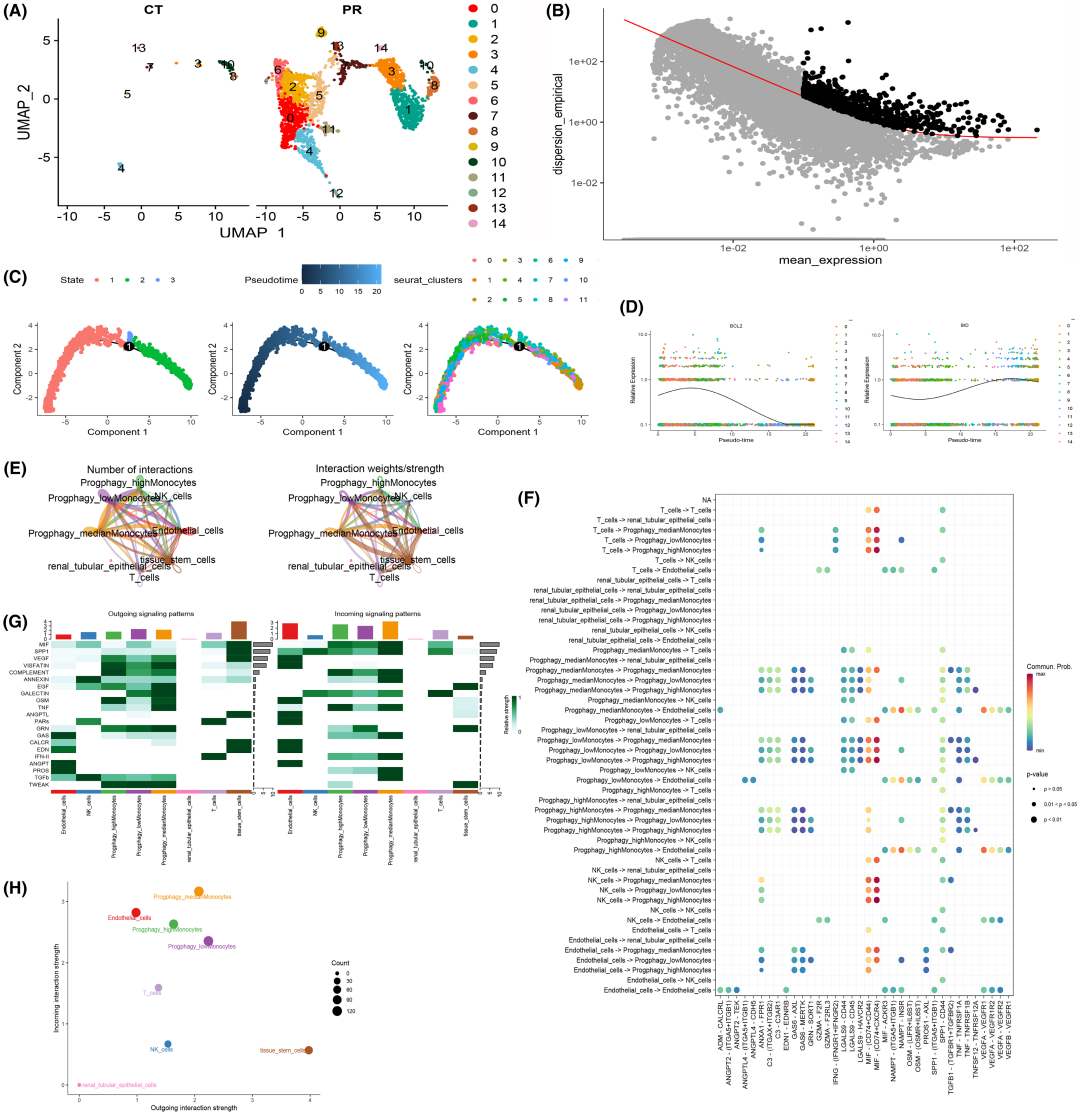
We performed pseudo‐timing and cell communication analyses in monocytes. (A) We extracted monocytes and divided 17‐PCDRGs into 14 subpopulations and (B) used ‘monocle’ to screen for hypervariable genes. (C) Trajectories and distributions of different monocyte subsets. (D) Temporal expression of *bcl2* and *bid* genes in the normal and disease groups. (E) We analysed the communication between three subsets of monocytes, as well as NK cells, renal tubular epithelial cells, endothelial cells, T cells and Tissue stem cells. (F) Bubble plot of the communication results. (G) Heatmap of signalling pathways. (H) Bubble maps showed that different subsets of monocytes had significant differences in MIF ‘incoming’ and ‘outgoing’.

Monocytes, a pivotal cell type, were judiciously isolated and methodically stratified into 14 subgroups predicated on the expression patterns of the 17 PCDRGs (Figure 11A). To unearth inherent patterns of gene expression variation, the identification of high‐variability genes was meticulously undertaken via the ‘monocle’ algorithm (Figure 11B). Our scrutiny of cell trajectory and pseudo‐time, particularly within this salient cell type, revealed a discernible colour spectrum signifying chronological progression. Cells exhibited an intricate developmental path, denoted by various shades of blue, corresponding to their progressive differentiation over time. Within the domain of monocyte cells, three distinct differentiation nodes were observed, each followed by cellular states indicative of other developmental phases. This intricate landscape elucidated the nuanced trajectories and divergent distribution patterns manifest across diverse monocyte subgroups (Figure 11C).In a subsequent phase, a profound exploration of temporal gene expression patterns came into focus. We meticulously evaluated the time‐dependent expression of the *bcl2* and *bid* genes, conducting a comprehensive comparative analysis between normal and disease groups. The findings accentuated the robust differential expression of a majority of PCDRGs over the course of this temporal series, a phenomenon that harmoniously corroborated our initial predictions (Figure 11D). Moreover, the monocyte cell population was further dissected into three distinct subgroups predicated upon variations in the expression levels of the 17 PCDRGs. Employing the ‘CellChat’ R package, we meticulously scrutinized the intricate landscape of intercellular communication that unfolded among these three subpopulations and their counterparts, including NK cells, renal tubular epithelial cells, endothelial cells, T cells and tissue stem cells. The tapestry of these communication interactions was both quantified and visualized, rendering insight into the extent of cellular exchanges (Figure 11E). The complex web of communication data was then seamlessly mapped onto the expansive human protein–protein interaction network, providing a comprehensive view of interactions between diverse cell populations. This network was adeptly distilled and presented in the form of informative bubble charts, effectively encapsulating the interplay between cell communities (Figure 11F).To delve deeper into the dynamics of this communication network, we calculated centrality metrics, thereby painting a comprehensive picture of signal pathway‐related communication strength among cell populations. We meticulously distinguished the origins and destinations of these communications, classified as ‘outgoing’ and ‘incoming’, respectively. Particularly noteworthy were the pronounced variations observed in factors such as MIF, GALECTIN, VISFATIN, CXCL and TNF among these three subgroups of monocyte cells (Figure 11G). The synthesis of these findings was elegantly encapsulated in the form of bubble charts generated using the netAnalysis_signalingRole_scatter function. Notably, the influence of distinct PCDRG expression profiles on monocyte cell communication, and by extension, on immune functionality, was vividly substantiated (Figure 11H).

### 3.11 CSF2 plays a key role in preventing the proliferation and migration of renal clear cell carcinoma cells

**FIGURE 12:**
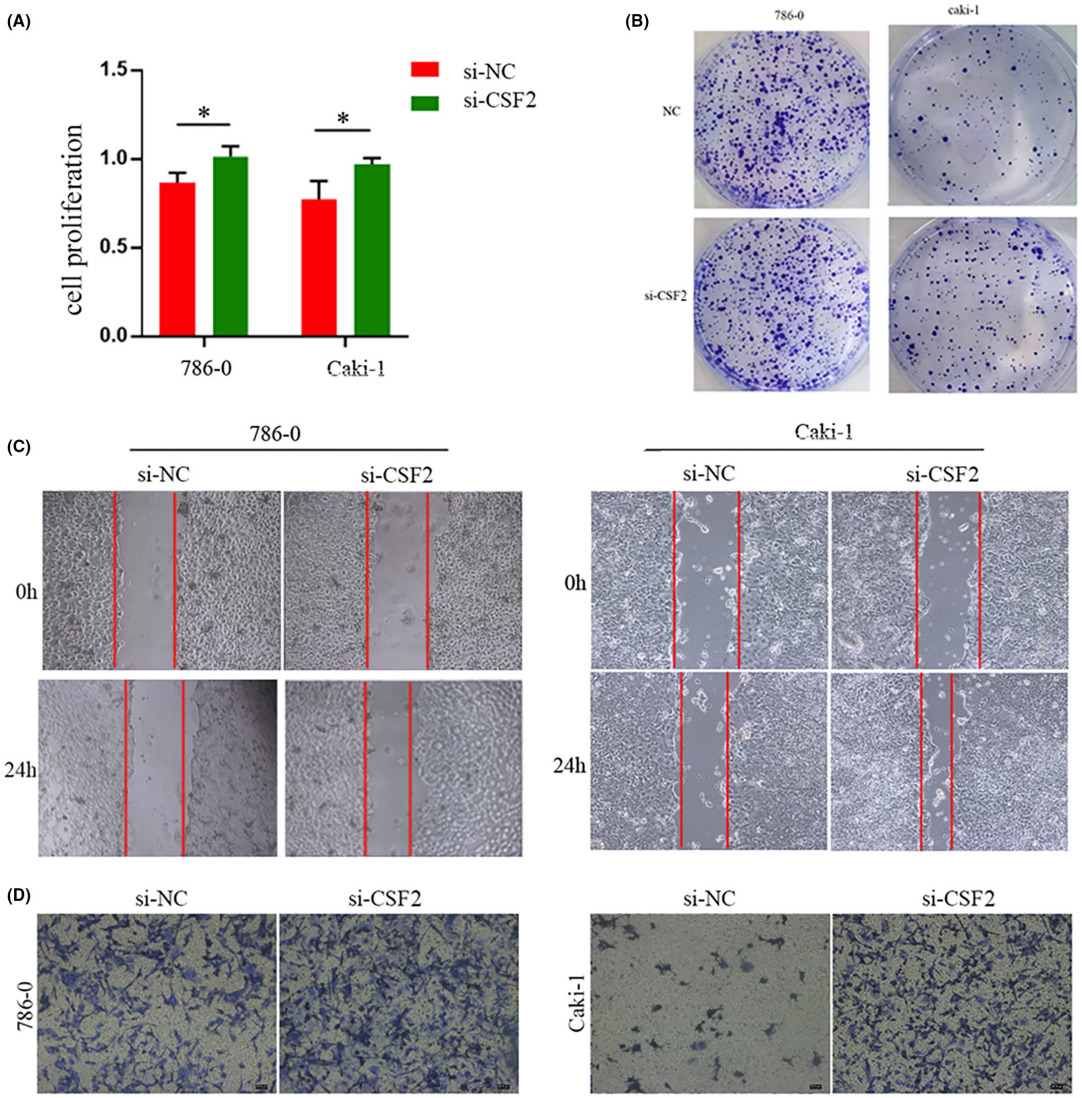
*csf2* has been shown to inhibit the growth, infiltration and movement of cells associated with renal clear cell carcinoma, as indicated by various analyses:(A) CCK‐8 analysis, (B) plate cloning assay, (C) wound healing assay and (D) transwell examination.

To thoroughly explore the potential involvement of *csf2* in renal clear cell carcinoma, a series of in vitro experiments was conducted. Notably, the CCK‐8 assay revealed a significant increase in cellular proliferative capacity upon silencing *csf2* (Figure 12A). The plate cloning assay substantiated that diminished *csf2* expression enhanced the proliferation and invasion potential of tumour cells (Figure 12B), while the scratch assay results illuminated the facilitative role of heightened *csf2* expression in tumour cell migration (Figure 12C). Subsequent findings from transwell assays underscored the consequential increases of cell invasion and migration capabilities following interference with *csf2* expression (Figure 12D). Taken together, these observations unveil the oncogenic properties associated with *csf2*, elucidating its pivotal role in preventing the proliferation, invasion, and migration of renal clear cell carcinoma.

## 4 DISCUSSION

ccRCC is an aggressive kidney neoplasm that originates from the clear cells inhabiting renal tubules. It represents a predominant subtype of kidney cancer, accounting for 85%–90% of malignant renal tumours.^24^, ^25^ If not promptly managed, it can exhibit aggressive growth, infiltrating adjacent tissues and organs, and even disseminating to distant sites such as the lungs, bones, and brain. In its advanced stages, ccRCC poses a substantial threat to overall health. Moreover, this malignancy frequently precipitates severe complications, encompassing hypertensive disorders, impaired renal function, and a spectrum of systemic ailments, significantly encumbering the patient's quality of life.^26^ Consequently, the quest for novel biomarkers assumes paramount importance, offering guidance for clinical management and augmenting prognostic precision.

Intriguingly, programmed cell death mechanisms emerge as pivotal orchestrators in tumorigenesis. Malignant cells, along with the stromal milieu, often circumvent the host's immune defences through the expression of immunosuppressive ligands, such as programmed cell death‐ligand 1 (PD‐L1). Programmed cell death plays a key role in renal clear cell carcinoma, influencing cancer initiation, progression and treatment response. In ccRCC, overexpression of anti‐apoptotic proteins such as Bcl‐2 and Bcl‐xL inhibited apoptosis and promoted the survival of tumour cells.^27^ p53 is an important tumour suppressor gene whose mutation or loss of function is common in various cancers, including ccRCC, which leads to a weakening of apoptosis signalling.^28^ Therefore, reinstating or augmenting apoptotic pathways holds profound therapeutic potential in the realm of cancer intervention.^29^ Furthermore, chemokines and their cognate receptors wield remarkable influence over diverse pathologies. Their purview extends beyond the immune domain, encompassing critical roles in tumorigenesis, including tumour growth, metastasis, and development.^30^ In light of these considerations, our investigation harnessed mRNA expression data from the TCGA‐KIRC dataset to discern pivotal prognostic genes and construct a multifaceted biomarker‐based prognostic model rooted in ccRCC‐related genes.

Programmed cell death assumes a pivotal role in the intricate orchestration of multicellular organisms' equilibrium. Across the spectrum of normal developmental processes, physiological contexts, and pathological states, PCD stands as the sentinel mechanism responsible for the selective elimination of cells predisposed to malignant transformation or those besieged by invasive pathogens. Notably, immune checkpoint inhibitors directed against the programmed cell death‐1 receptor (PD‐1) have exhibited the capacity to enhance the survival outcomes in a subset of ccRCC patients.^31^ In light of these insights, our investigation unfolds with a deliberate focus on a comprehensive array of 15 distinct PCD modalities, encompassing apoptosis, pyroptosis, ferroptosis, autophagy, necroptosis, cuproptosis, parthanatos, entotic cell death, netotic cell death, lysosome‐dependent cell death, alkaliptosis, oxeiptosis, disulfidptosis, anoikis and efferocytosis.

While antecedent investigations have probed various facets of these processes, a prevailing consensus emerges, suggesting elevated expression profiles of PCD‐associated genes within KIRC tissues compared to their non‐neoplastic counterparts.^32^ Nonetheless, it is pertinent to underscore that these earlier inquiries primarily traversed isolated aspects of PCD, thus signifying the pressing need for a holistic interrogation of their interconnected dynamics in the context of ccRCC. It has been shown that drugs that promote ferroptosis, such as siderophores and lipid peroxides generating agents, can enhance the sensitivity of ccRCC cells to treatment.^33^

This study, in pursuit of a rigorous investigation, amalgamated gene expression data pertaining to PCDRGs from the TCGA‐KIRC dataset. From this repository, 17 pivotal genes were meticulously culled, each carrying substantive import, and were identified as *bcl2*, *bcl3*, *bid*, *cdkn1a*, *chac1*, *csf2*, *cx3cl1*, *bdh*, *ddias*, *erbb3*, *il4*, *ltbr*, *pdcd5*, *pink1*, *plaur*, *ybx3* and *prkag3*. Through the assimilation of this gene cohort, we embarked on the conception of a novel PCDRG‐based prognostic model, a synthesis derived from the confluence of 10 distinct machine learning methodologies, yielding a total of 101 models. Additionally, the formidable framework of Cox proportional hazards regression analysis was employed, rendering a composite architecture underpinning the prognostic paradigm.

In delving into the ontological framework of these genes, it becomes evident that they are intrinsically interwoven with various facets of cell death modalities. Akin to orchestral players in a symphony, these genes lend their distinct notes to the overarching composition of cell fate. *bcl2*, *bcl3*, *bid*, *cdkn1a*, *chac1*, *csf2*, *cx3cl1*, *bdh*, *ddias*, *erbb3*, *il4*, *ltbr*, *pdcd5*, *pink1*, *plaur* and *ybx3* are intricately associated with the intricate orchestration of apoptosis. *chac1* is deeply implicated in the orchestration of ferroptosis, while *bcl2*, *il4*, *pink1* and *prkag3* find their mooring in the landscape of autophagy. The solemn domains of necroptosis welcome *bcl2* and *bid*, while *il4* casts its gaze upon lysosome‐dependent cell death, and *bcl2* is a sentinel guardian in the realm of anoikis.

The laudable precedent set by earlier research corroborates the pertinacious involvement of these genes in the intricate tapestry of tumorigenesis and cancer pathogenesis. As an illustrative case in point, the intimate nexus between NF‐KATAB and BCL2 in ccRCC has not eluded the discerning gaze of scholars, intimating a compelling case for the effectiveness of NF‐KATAB as a therapeutic agent.^34^ BCL‐2's intricate choreography with the pro‐apoptotic conductor Bax assumes the role of a cell apoptosis regulator in ccRCC.^35^ The downregulation of Bcl3 emerges as an inhibitory force in ccRCC, exerting its influence over metabolic and vascular processes, as documented.^36^ In the theatre of apoptosis, *bid* takes center stage, with its malfunction forming the underpinning for oncogenic development and emerging as a credible risk factor in the annals of ccRCC.^37^ A previous study found that the emergence of *cdkn1a*'s antisense DNA damage‐activated RNA promoter as a conspicuous presence in tumour tissues renders it a key protagonist, additionally serving as an independent harbinger of OS in ccRCC.^38^ The immunoregulatory gene *cx3cl1* emerges as a prominent constituent in the inflammatory milieu of ccRCC, bestowing a pathway to personalized treatment strategies.^39^ *ddias*, when overexpressed in NCI‐H1299 or NCI‐H1703 cells, assumes an inhibitory role, counteracting the proliferation induced by CHIP overexpression. This finding underscores the substantive pertinence of CHIP‐dependent *ddias* instability in the domain of cancer cell growth.^40^ The genetic locus of *erbb3* is unveiled as a significant determinant, embracing ccRCC and papillary RCC under its encompassing aegis.^41^ Among the diverse pantheon of genes, high expression levels of *ybx3* stand in a significant nexus with the pathological staging of tumours, histological grading, TNM staging, and the abundance of immune cell subpopulations. These findings ensconce *ybx3* in the progression and prognosis milieu of ccRCC.^42^

Following an intricate analytical odyssey, entailing both univariate and multivariate COX scrutiny, the meticulously crafted PCDRG profile emerges as a formidable and autonomous prognostic determinant in the complex landscape of ccRCC. This predictive prowess is further underscored by the categorical division of patients into two discrete prognostic subgroups, demarcated by the median risk score. The resultant delineation paved the way for the development of robust survival curves, ROC curves, nomogram curves and calibration curves.

Upon a comprehensive evaluation of the ensemble of analytical outputs, it becomes apparent that the PCDRG profile wields a predictive influence of significant magnitude in shaping the destiny of ccRCC patients. This predictive acumen surpasses that of established clinical parameters, including age, gender, histological stage and tumour grade. The concordance between the predictive values and those observed in the clinical realm reinforces the theoretical underpinnings, bequeathing healthcare professionals with a dependable compass for clinical diagnosis and patient management.

Our exploration revealed distinctive patterns of immune infiltration within the context of the model genes, suggesting their multifaceted roles in renal clear cell carcinoma that span diverse programmed cell death processes.

In the realm of apoptosis, the pro‐apoptotic BCL‐2 family member, Bim, emerges as a noteworthy tumour suppressor, particularly in neoplastic mast cells. It assumes a downregulated disposition, primarily influenced by the oncogenic kinase KITD816V's activity, upon stimulation by the stem cell factor (SCF).^43^ In the wake of SCF‐mediated upregulation, a cascade of effects ensues, contributing to the secretion of immune‐modulating entities like transforming growth factor (TGF)‐β and interleukin (IL)‐10. A previous study found that this molecular environment promotes the establishment of an immunosuppressive tumour microenvironment that strategically orchestrates the evasion of immune surveillance and responses within the tumour.^44^ Apoptosis CSF2 belongs to this pattern of programmed cell death. Cancer cells escape from apoptosis and continue to proliferate by dysregulated apoptotic signalling pathways, such as downregulation of death receptors (e.g. Fas, TNFR) or up‐regulation of inhibitory molecules (e.g. FLIP) through extrinsic apoptotic pathways.^45^ In addition, renal cancer cells also prevent cell apoptosis by directly inhibiting the activity of Caspases (apoptosis executing enzymes).^46^

Autophagy, on the other hand, casts its influence through the modulation of *pink1*, which significantly impacts critical immune cell components, such as NK cells and CD8^+^ T cells. This modulation extends its reach to key chemotactic processes mediated by MHC‐I. The role of cytotoxic T cells, bearing surface CD8, is pivotal in the arena of anti‐cancer immunotherapy, including the intricate domain of kidney renal clear cell carcinoma (KIRC).^47^ Further examinations unveiled pronounced distinctions in the expression of *pink1* within these immune cell types, differentiating high‐risk and low‐risk groups. We observed notable distinctions in monocyte expression profiles between the high‐risk and low‐risk groups, reflecting an intricate interplay within the tumour microenvironment of KIRC. These disparities align with prior findings, indicating escalated monocyte cellular senescence in the tumour microenvironment compared to normal tissues.^48^

Crucially, immune function intricacies involve the chemokine receptor CCR, significantly influencing monocyte functionality. Notably, CCR2, characterized by its widespread presence in monocytes and affinity for its specific ligand, MCP‐1, has garnered considerable attention. Experimental studies employing murine models demonstrated that the blockade of the CCL2/CCR2 axis exerts substantial impacts. This intervention reduced the recruitment of inflammatory monocytes and macrophages, notably from primary tumour sites and pre‐metastatic liver niches. Consequently, these effects culminate in bolstered anti‐tumour immunity, instigate tumour regression, and impede the metastatic cascade. Thus, the CCL2/CCR2 signalling pathway emerges as a promising avenue for therapeutic exploration in the context of kidney cancer, warranting dedicated research.^49^ Monocytes wield their influence through the release of a spectrum of pro‐inflammatory mediators, including TNF‐α, IL‐1β, IL‐6, MCP‐1, CCL2, IL‐8 and CXCL8. Furthermore, extrinsic cell apoptosis pathways are activated through the ligation of death receptors, constituting a subset of the TNF receptor (TNFR) superfamily.^50^ Insight gleaned from the analysis of OTUD1 RNA‐seq data elucidates a potential nexus involving OTUD1 inhibition and the AKT and NF‐κB pathways in kidney cancer cells. Notably, OTUD1 engages in interactions with PTEN, impacting PTEN stability and exerting regulatory influence.^51^

Notably, inflammasome activation serves as a trigger for the release of interleukin‐1β (IL‐1β) and IL‐18, which in turn propagate pyroptotic cell death processes.^52^ Notably, CXCL7 assumes a pivotal role in driving cell proliferation, both in in vivo and in vitro settings, and its expression is modulated by the central pro‐inflammatory cytokine interleukin‐1β. This is pertinent, as ccRCC cells generally exhibit low levels of CXCL7, yet its expression becomes notably elevated within the tumour milieu due to heightened IL‐1β levels.^53^

Furthermore, our exploration ventured into the realm of drug sensitivity, a crucial aspect with discernible differences observed between the high‐risk and low‐risk groups (*p* < 0.05). For instance, rapamycin (*p* = 0.01) manifested heightened sensitivity within the high‐risk group. These observations assume significance in the context of treatment options for advanced kidney cancer, particularly ccRCC patients. These options encompass vascular endothelial growth factor (VEGF) inhibitors and mammalian target of rapamycin (mTOR) pathway inhibitors.^54^ Noteworthy is the heightened sensitivity exhibited by the chemotherapy drug vinblastine within the high‐risk group (*p* = 0.05). Importantly, clinical applications involving recombinant interleukin‐2 in combination with vinblastine have demonstrated notable efficacy in the treatment of metastatic kidney cell carcinoma.^55^

Our utilization of single‐cell technology has conferred significant advantages upon the analysis of the immune microenvironment within pathological tissues at the cellular level, as emphasized in recent literature.^56^ Furthermore, our approach, underpinned by single‐cell sequencing data, facilitated the execution of pseudotime analysis. This methodology approximates real‐time trajectories, effectively mirroring the dynamic alterations in PCDRG expression and cellular differentiation throughout the course of disease progression.

Our in‐depth exploration of GO enrichment analyses revealed a notable surge in gene enrichment, particularly emphasizing the significance of GO:0009897. This functional annotation pertains to the peripheral adhesive complex, a pivotal molecular player bridging cellular adhesion, cell–cell interactions, and cell‐matrix interactions, which has been extensively detailed in prior research.^57^ In the light of these findings, we conducted a more comprehensive investigation focusing on the interplay among monocytes.

Of particular interest is the molecular involvement of Macrophage Migration Inhibitory Factor (MIF), renowned for its status as a non‐homologous ligand binding to chemokine receptors CXCR2, CXCR4 and CXCR7. MIF takes on the role of a chemokine‐like molecule, orchestrating monocyte activation via intricate Galpha i‐dependent signalling cascades and integrin‐mediated adhesion processes, ultimately culminating in monocyte recruitment.^58^

The Von Hippel–Lindau (VHL) gene, distinguished for its multifaceted roles, primarily functions as an E3 ubiquitin ligase. It exerts strict control over the stability of hypoxia‐inducible factor subunits HIF‐1α and HIF‐2α, and this regulation occurs in an oxygen‐dependent fashion. In the context of renal cancer, the loss of VHL triggers the constitutive activation of HIF transcriptional complexes. This cascade leads to the overexpression of classical HIF target genes such as GLUT1, VEGF and CAIX (carbonic anhydrase IX). Importantly, MIF emerges as a direct target gene of HIF. Notably, the receptor for MIF, CD74, exhibits heightened expression levels in ccRCC.^59^ Our explorations have brought to light that the most pronounced MIF expression, particularly CD74^+^CXCR4, characterizes the intercellular communication dynamics involving renal tissue stem cells and monocytes.

Collectively, these revelations underscore the potential for tailoring immunotherapies, capitalizing on patient‐specific expression profiles of PCDRGs and immune infiltration. Such precision‐oriented approaches hold the promise of yielding superior clinical outcomes.

Indeed, experimental results showing that CSF2 inhibits the growth, infiltration, and movement of renal clear cell carcinoma‐associated cells may reflect its complex role in regulating immune responses and the tumour microenvironment. Specifically, CSF2 may promote immune surveillance and clearance of tumours by enhancing the activity of immune cells, such as monocytes and dendritic cells. In addition, CSF2 may influence tumour growth and infiltration by regulating the differentiation and proliferation of tumour‐associated cells. Due to the tumour heterogeneity and complexity of renal clear cell carcinoma. The results of the bioconfidence analysis may reflect the overall trend of high CSF2 expression in tumour tissues, whereas the experimental results reveal more directly the role of CSF2 in specific cell types and conditions. Therefore, further studies are needed to explain this contradiction and to better understand the mechanism of CSF2's role in renal clear cell carcinoma.

## 5 CONCLUSION

Our study represents a significant leap forward in the understanding and clinical management of KIRC. By unravelling a repertoire of PCDRGs and crafting a bespoke prognostic model tailored for ccRCC, we have illuminated new avenues for improving patient outcomes and refining therapeutic approaches. Through the integration of prognostic models based on PCDRGs, clinicians can tailor therapies to the specific genetic makeup and disease characteristics of each patient. This individualized approach promises to revolutionize ccRCC treatment, offering improved outcomes and quality of life for affected individuals. By elucidating the intricate interplay between PCDRGs and the immune microenvironment, we have uncovered novel targets for immunotherapeutic interventions. In summary, our study not only expands our understanding of ccRCC but also translates this knowledge into tangible clinical benefits. From drug screening to personalized therapy and immunotherapy, the potential applications of our findings are vast and far‐reaching. We are optimistic that our research will serve as a catalyst for the development of more effective therapeutic interventions, ultimately improving outcomes for patients with ccRCC.

## AUTHOR CONTRIBUTIONS

**Hao Chi:** Conceptualization (equal); writing – review and editing (equal). **Hongtao Tu:** Data curation (equal); formal analysis (equal); visualization (equal); writing – original draft (equal). **Qingwen Hu:** Data curation (equal); formal analysis (equal); visualization (equal); writing – original draft (equal). **Yuying Ma:** Data curation (equal); formal analysis (equal); validation (equal); visualization (equal); writing – original draft (equal). **Jinbang Huang:** Formal analysis (equal); visualization (equal); writing – original draft (equal). **Honghao Luo:** Data curation (equal); writing – original draft (equal). **Lai Jiang:** Formal analysis (equal); visualization (equal); writing – original draft (equal). **Shengke Zhang:** Writing – original draft (equal). **Chenglu Jiang:** Writing – original draft (equal). **Haotian Lai:** Writing – original draft (equal). **Jie Liu:** Writing – original draft (equal). **Jianyou Chen:** Writing – original draft (equal). **Liwei Guo:** Writing – original draft (equal). **Guanhu Yang:** Data curation (equal). **Ke Xu:** Conceptualization (equal); writing – review and editing (equal). **Haiqing Chen:** Conceptualization (equal); writing – review and editing (equal).

## FUNDING INFORMATION

The study was approved by Dazhou Science and Technology Bureau project (21ZDYF0028) and Dazhu Science and Technology Bureau project (2022NCG0013 and 2022NCG0014).

## CONFLICT OF INTEREST STATEMENT

The authors declare that they conducted this study free from any current or potential commercial or financial affiliations that could create a conflict of interest.

## Supporting information


Table S1.

## Data Availability

The datasets utilized in our investigation are accessible via the GEO repository (https://www.ncbi.nlm.nih.gov/geo/) and the TCGA database (https://portal.gdc.cancer.gov/). The unprocessed data can be obtained from jianguoyun at the following link: https://www.jianguoyun.com/p/De3gBZQQt9WQDBiI4MIFIAA.
